# The global need for essential emergency and critical care

**DOI:** 10.1186/s13054-018-2219-2

**Published:** 2018-10-29

**Authors:** Carl Otto Schell, Martin Gerdin Wärnberg, Anna Hvarfner, Andreas Höög, Ulrika Baker, Markus Castegren, Tim Baker

**Affiliations:** 1 Global Health—Health Systems & Policy, Department of Public Health Sciences, Karolinska Institutet, Stockholm, Sweden; 2 Centre for Clinical Research Sörmland, Uppsala University, Uppsala, Sweden; 3 Faculty of Medicine, Uppsala University, Uppsala, Sweden; 4 College of Medicine, Blantyre, Malawi; 5 Perioperative medicine and intensive care (PMI), Karolinska University Hospital, Stockholm, Sweden

**Keywords:** Critical care, Emergency care, Health services, Quality of care, Critical illness, Global health, Developing countries, Universal health coverage, Patient safety

## Abstract

Critical illness results in millions of deaths each year. Care for those with critical illness is often neglected due to a lack of prioritisation, co-ordination, and coverage of timely identification and basic life-saving treatments. To improve care, we propose a new focus on essential emergency and critical care (EECC)—care that all critically ill patients should receive in all hospitals in the world. Essential emergency and critical care should be part of universal health coverage, is appropriate for all countries in the world, and is intended for patients irrespective of age, gender, underlying diagnosis, medical specialty, or location in the hospital. Essential emergency and critical care is pragmatic and low-cost and has the potential to improve care and substantially reduce preventable mortality.

## Introduction

“Help! This patient is very sick, we must do something immediately!”; a frequently heard cry in hospitals all over the world. Such patients are “critically ill”. The term is commonly used, and yet its definition is elusive. Using a working description of “any immediately life-threatening, reversible condition”, critical illness is the most severe stage of acute illness and, if left untreated, often leads to a poor outcome or death [1]. Critical illness can occur in anyone irrespective of age, gender, or social status, it can begin in the community or in hospital, and does not respect traditional divisions into medical specialties. Patients with conditions such as sepsis, pneumonia, eclampsia, haemorrhage, trauma, peritonitis, asthma, and stroke can all have or develop critical illness and, as such, critical illness has been crudely estimated to result in several million deaths globally each year [2]. In this article, we propose a new concept of essential emergency and critical care (EECC), defined as “the care that all critically ill patients should receive in all hospitals in the world”.

### Emergency and critical care

Patients with critical illness require care and require it quickly. This is “emergency and critical care”, the identification and continued observation, assessment, and treatment required to manage critical illness [3]. Emergency and critical care focuses on resuscitating unstable patients and allowing time for recovery or the effect of specific therapies to improve outcomes and prevent death. We use emergency and critical care in the broad sense of care provided to *all* critically ill patients. Emergency and critical care is therefore for those who are critically ill at arrival, or who were stable and subsequently deteriorated, and can be provided anywhere in the hospital: in the emergency department, the intensive care unit (ICU), general wards, post-operative recovery units, and high-dependency units [2, 3].

### Neglected emergency and critical care

**Fig. 1:**
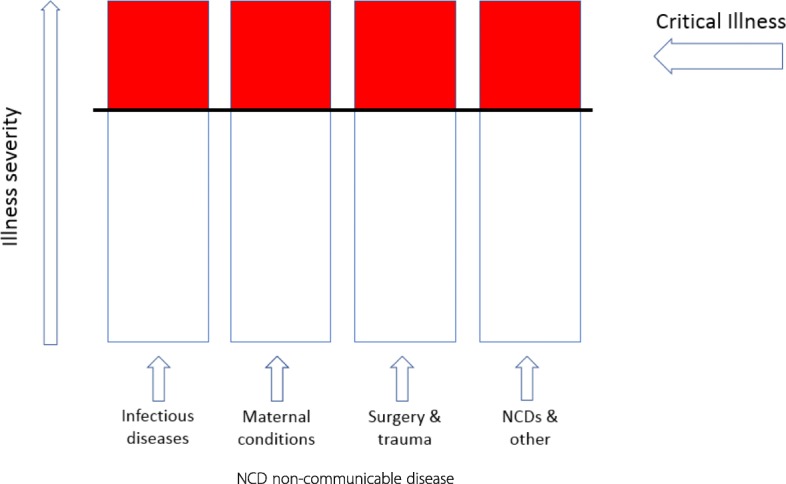
Critical illness: a horizontal illness-severity perspective

There are several reasons to suspect that emergency and critical care may be neglected. Firstly, the approach used by most medical specialties and health systems is typically “vertical”, meaning that diagnosis-related and specialty-related care are prioritised rather than the severity-related “horizontal” approach of emergency and critical care (Fig. 1). Moreover, in hospitals in low-resource settings and in general wards in high-resource settings, there can be low staffing levels, a lack of equipment, and limited knowledge or awareness of emergency and critical care which can result in a failure to identify critical illness and an under-prioritisation of emergency and critical care [4–7]. Secondly, there is a lack of commonly agreed definitions and criteria for either the identification of critical illness, or for the provision of emergency and critical care. Thirdly, emergency and critical care can be conflated with technologically advanced and expensive care on ICUs. ICUs have spread worldwide and developed rapidly since their origin in the 1950s and are now specialised units managing patients with multi-organ failure [2, 3, 8–10]. ICU treatment is expensive and can cost up to 1% of the economy of a high-income country [11], and is thus rationed. As the number of ICU beds per million population varies substantially—from 292 in Germany, 58 in Sweden, 25 in Sri Lanka, to 1 in Uganda [10, 12]—it is clear that ICUs cannot be the sole providers of care for all critically ill patients. Finally, it can be presumed that the acute care provided at arrival to hospital is sufficient, and deteriorating patients in the hospital can be missed. While a proportion of critically ill patients do receive emergency and critical care in emergency departments, in ICUs, or in other hospital settings, there are many who are not identified or do not receive the care that they so urgently need.

The consequences can be poor quality care for critically ill patients. The co-ordination of emergency and critical care may be difficult within a hospital and within the health system. The basic, life-saving therapies that form the core of emergency and critical care may be forgotten. A focus on expensive ICU beds rather than broader emergency and critical care may drain valuable resources, treating a few individuals at a high cost instead of many critically ill at a low cost, and a focus on acute care in emergency departments can exclude hospital in-patients. The likely result is substantial preventable mortality and morbidity.

### Essential emergency and critical care

To improve the care of the critically ill, we propose a new focus on “essential emergency and critical care” (EECC). We define EECC as “care that all critically ill patients should receive in all hospitals in the world”. EECC is a concept that should be a crucial part of universal health coverage; it is low-cost and appropriate for all countries in all settings in the world, and is intended for patients irrespective of age, gender, underlying diagnosis, medical specialty, or location. EECC is a set of actions and treatments plus the system-wide requirements for their provision. For simplicity of definition, we limit EECC to care in hospitals, rather than in primary care clinics or the pre-hospital environment.

**Fig. 2:**
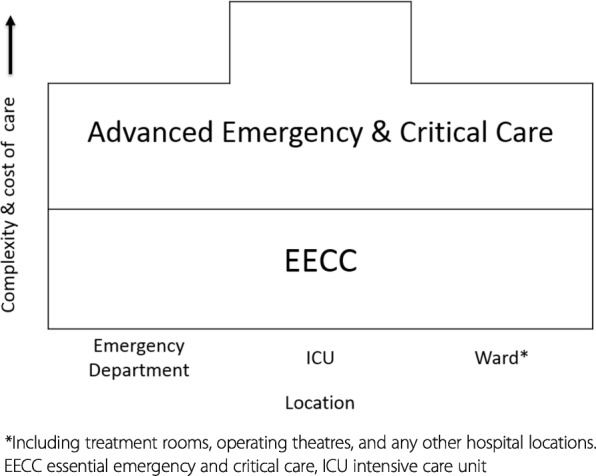
Relationship between EECC, advanced emergency and critical care, and location in hospitals

As the critically ill are a heterogeneous group it is important to define what is *not* included in EECC. It is not the diagnosis and definitive treatment that focus on the pathophysiological cause of the critical illness—actions which are also important for the patient—and it is not the “advanced emergency and critical care” that may also be available in high-resource settings (Fig. 2). EECC is the low-cost, most basic level of emergency and critical care and should be provided in *all* settings. The available resources will determine what additional care is appropriate or possible. EECC is not the palliative care required for dying patients, or care when a patient’s condition is so severe that recovery has been deemed impossible. While the decision to provide palliative care instead of EECC can be very challenging, it is nevertheless of great importance and the adoption of EECC must not be allowed to lead to increased suffering for patients for whom there is no hope of a good recovery.

**Fig. 3:**
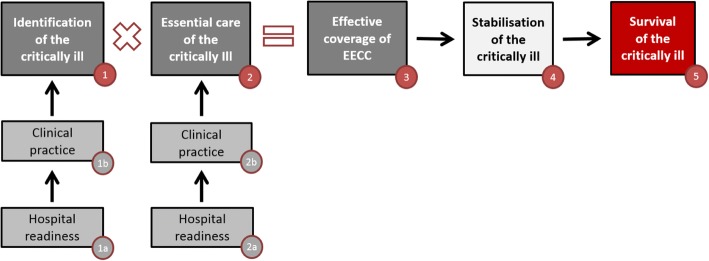
Essential emergency and critical care (EECC) in hospitals: a conceptual framework. The numbers in circles correspond to the text in Table 1

EECC can be visualised in a framework (Fig. 3), and the elements of EECC are described in Table 1. EECC is divided into two key domains: identification and care. To identify a case and to provide care, “hospital readiness” (the necessary facilities or structures in the hospital) is required. Subsequently, “clinical practice” (the processes of care) is required. The product of the identification and care is the output, or “effective coverage” [13], of EECC: the proportion of all critically ill patients in hospital who receive EECC. Our hypothesis is that a high effective coverage of EECC leads to good outcomes and survival for many critically ill patients.

**Table 1:** The elements of essential emergency and critical care (EECC) in hospitals

1	Identification of the critically ill: the proportion of critically ill patients who are identified		
	1a	The structures needed for a hospital to have the potential to identify the critically ill	
		For example:	
			Emergency department (ED) triage system
			Ward-based triage
			Trained ED and ward staff
			Pulse oximeter
	1b	The clinical processes needed for the identification of the critically ill	
		For example:	
			ED triage is conducted
			Ward triage, for example early warning score (EWS), is conducted
2	Essential care of the critically ill: the proportion of those identified as critically ill who receive essential care		
	2a	The structures needed for a hospital to have the potential to provide essential care of the critically ill	
		For example:	
			Availability of ED resuscitation room
			Emergency drugs and equipment
			Oxygen
			Trained staff
			Guidelines for EECC
	2b	The clinical processes needed for essential care of the critically ill	
		For example:	
			Use of appropriate airway actions
			Use of oxygen in hypoxia
			Use of intravenous fluids in shock
3	The proportion of all critically ill patients who receive EECC: the output of EECC		
	For example:		
		If 50% of all critically ill patients in a hospital are identified and 80% of these receive the correct essential care, then effective coverage of EECC is 40%	
4	The mechanism through which EECC translates into increased survival of the critically ill		
	For example:		
		Airway maintained	
		Breathing supported	
		Circulation maintained	
5	The desired outcome of EECC: survival of the critically ill		
	For example:		
		To a defined time point; for example, hospital discharge	

The examples are elements that could be included in EECC

The principles underlying EECC are not new. Florence Nightingale in the 1850s described more frequent observations of the sickest patients and moved them closest to the nurses’ station, and the first ICUs opened 60 years ago [3]. The specialties of emergency medicine and intensive care medicine have the principles of EECC as core competencies, and several modern initiatives implicitly utilise EECC. Triage systems in emergency departments categorise patients into levels of urgency and may specify the care required for patients at each triage level [14]. Vital sign-based early warning scores and treatment protocols [15–17], rapid response teams of ICU outreach staff [18, 19], and initiatives for improving ward care [20, 21] aim to improve processes for the identification and care of sick hospital patients. EECC can provide a unifying concept for such initiatives, ensuring a system-wide emphasis on illness severity and prioritisation for those at highest risk so that the most fundamental care for the critically ill is delivered throughout the hospital. EECC has global relevance: in a low-resource setting, EECC may entail directing scarce human resources towards the sickest patients and ensuring limited medicines and equipment are used where they could have the biggest impact; in a high-resource setting, EECC may ensure that vertical, specialist services do not neglect the identification and care of critically ill patients.

The idea of “essential” services has been used in other disciplines. The World Health Organization (WHO) has had an essential medicines list since 1977 [22]. The WHO guidelines for essential trauma care are defined as “achievable standards for trauma treatment services which could realistically be made available to almost every injured person in the world” [23]. The WHO Guide to Essential Practice for Pregnancy, Childbirth, Postpartum and Newborn Care is now in its third edition [24]. The word “essential” is used to define a minimum set of actions that should always be implemented. It is a conceptual way of “raising-the-bottom”, in contrast to frequently used “lifting-the-top” approaches.

### Impact

What would be the impact of improving the effective coverage of EECC? Hospitals providing EECC to all their patients would have a system-wide approach for managing critical illness, an approach that could prevent deterioration and save lives at a low cost. Critically ill patients would be identified and treated quickly throughout the hospital. Critical care gaps that exist between the emergency department and the wards, between the ICU and the wards, and between specialties would be closed. No patient would die from a condition that EECC could prevent.

### The way forward

To operationalise EECC, we propose the following research and policy agenda. Firstly, clear definitions of critical illness and emergency and critical care should be established. Secondly, the specific values and contents of EECC should be defined in a transparent way, involving the opinions of diverse stakeholders and experts. Thirdly, a research agenda should be designed, evaluating the current effective coverage of EECC (see Fig. 3), the impact of EECC interventions, and the effectiveness of implementation strategies. Finally, medical educators, policy makers, and health system experts should be involved throughout the process to ensure policy relevance and buy-in, shortening the distance from research findings to curriculum design and policy implementation.

## Conclusions

We have described the concept of essential emergency and critical care (EECC). We believe EECC should be a crucial part of universal health coverage, and that EECC has the potential to improve the care given to critically ill patients in hospitals in all settings in the world and substantially reduce preventable mortality.

## Abbreviations

EECC: Essential emergency and critical care
ICU: Intensive care unit
